# Analysis of PET parameters predicting response to radiotherapy for myeloid sarcoma

**DOI:** 10.1371/journal.pone.0261550

**Published:** 2021-12-20

**Authors:** Kyu Hye Choi, Jin Ho Song, Yoo-Kang Kwak, Jong Hoon Lee, Hong Seok Jang

**Affiliations:** 1 Department of Radiation Oncology, Seoul Saint Mary’s Hospital, College of Medicine, The Catholic University of Korea, Seoul, Republic of Korea; 2 Department of Radiation Oncology, Incheon Saint Mary’s Hospital, College of Medicine, The Catholic University of Korea, Seoul, Republic of Korea; 3 Department of Radiation Oncology, Saint Vincent’s Hospital, chlrb2 College of Medicine, The Catholic University of Korea, Seoul, Republic of Korea; Spedali Civili of Brescia, University of Brescia, ITALY

## Abstract

**Purpose:**

Positron-emission tomography (PET)-CT has recently been used for diagnostic imaging and radiotherapy for myeloid sarcoma, but there is little research on predicting the response of radiotherapy. The aim of this study was to analyze the association between PET-CT variables and the response to radiotherapy in patients with myeloid sarcoma.

**Materials and methods:**

This study was conducted in myeloid sarcoma patients who received radiotherapy and PET-CT before and after radiotherapy. The response to radiotherapy was evaluated based on the European Organization for Research and Treatment of Cancer PET response criteria, and binary regression analysis was performed to assess the factors predicting reductions in the maximum standardized uptake value (SUVmax).

**Results:**

Twenty-seven sites in 12 patients were included in the study. Complete metabolic responses were seen in 24 patients after radiotherapy, a partial metabolic response in one, and progressive metabolic disease in two patients. The prescribed dose of more than 3000 cGy_10_ was significantly greater in the treatment control group (*P* = 0.024). In binary logistic regression analysis predicting reductions in the SUVmax of more than 70% after radiotherapy, the pretreatment SUVmax (≥ 7.5) and further chemotherapy after radiotherapy showed significant differences in univariate and multivariate analyses.

**Conclusion:**

Good metabolic responses (complete or partial) to radiotherapy were achieved in 92.6% of the myeloid sarcoma patients. Radiation doses < 3000 cGy_10_ and increased SUVmax were related to treatment failure and high SUVmax before radiotherapy was a factor influencing SUVmax reduction. Further large-scale studies are needed.

## Introduction

Myeloid sarcoma is a solid tumor formed by leukemic cells outside the bone marrow and was also called chloroma in the 19^th^ century because the tumors showed a greenish hue from the myeloperoxidase enzyme [1]. It usually occurs in patients with acute myeloid leukemia (AML) and other myeloproliferative diseases or myelodysplastic conditions, and rarely, in lymphoid leukemia. Tumors usually appear in bones, but can occur in any part of the body including the skin, and are often found in imaging studies such as computed tomography (CT) or magnetic resonance imaging (MRI) without symptoms in approximately 50% of the patients [2]. Patients with leukemia and myeloid sarcoma have a poor prognosis, so systemic therapy as treatment of choice even for localized myeloid sarcoma due to high rate of progression is recommended. However, local treatment is actively performed in symptomatic or life-threatening lesions with organ involvement [3–5]. Among the local treatments, radiation therapy is known to have a relatively good treatment response and symptom relief rate [6, 7].

Diagnosis of myeloid sarcoma will require histologic confirmation (either tissue biopsy or bone marrow biopsy) in myeloid sarcoma. If pathologic diagnosis cannot be performed, myeloid sarcoma can be diagnosed through CT or MRI. In radiotherapy planning, PET-CT helps to delineate the targets of radiotherapy and can be used to assess the response to therapy [8]. However, little is known about the usefulness of PET-CT in the treatment of myeloid sarcoma, and no research has been conducted on whether it can be used to predict the outcome of radiotherapy.

The purpose of this study was to analyze the relationship between the parameters of PET-CT and the treatment response of myeloid sarcoma patients treated with radiotherapy and identify the potential outcome predictors of radiation therapy.

## Materials and methods

This study was a retrospective study of patients who received radiation therapy for myeloid sarcoma and PET-CT before and after treatment at Seoul St. Mary’s Hospital between March 2015 and August 2019. Prior to treatment, the patient’s diagnosis, previous bone marrow transplantation (BMT), and total body irradiation (TBI) history were reviewed through the medical records.

PET-CT was performed before radiotherapy, and the maximum standardized uptake value (SUVmax) before treatment and 3–6 months after the completion of radiotherapy were extracted and the difference was converted into a percentage. For radiotherapy, the target was delineated through PET-CT, then 5–10 mm was extended from the gross tumor volume (GTV) to the planning target volume (PTV). Both 3-dimensional conformal radiotherapy (3D-CRT) and intensity-modulated radiotherapy (IMRT) were included as radiotherapy techniques and the biologically effective dose (BED) was calculated for each case using an alpha/beta ratio of 10 to compare different dose schedules.

All patients fasted for at least 6 hours before the PET-CT scans and were in the supine position during scanning. A nonenhanced CT scan with a 5 mm slice thickness was performed, and the intravenous injection of 3.7–5.5 MBq/kg of ^18^F-fluorodeoxyglucose started the scan 60 minutes later. Images were acquired using a combined PET-CT in-line system with a Biograph Duo (Siemens Medical Solutions, Knoxville, TN, USA).

The criteria for the response to PET were set based on the European Organization for Research and Treatment of Cancer (EORTC) PET response criteria [9]. The Response Evaluation Criteria in Solid Tumors (RECIST) criteria in follow-up CT image was used for evaluation of response after completion of radiotherapy. For analysis of the treatment dose for radiation therapy, the radiation dose converted to BED with an alpha/beta ratio of 10 (BED10) and the volume of the lesion before treatment were extracted. Recurrence was defined as new lesion development during the follow-up period after the response to radiation therapy. The pattern of recurrence was investigated through imaging studies every 3–6 months after treatment. In the case of recurrence, the recurrence pattern was described in detail. The recurrence patterns were defined as follows. An in-field recurrence was when 95% of the recurrence volume was within the 90% isodose curve of the radiotherapy field, and a marginal recurrence was assigned when 20–95% of the recurrence volume was within the 90% isodose curve.

The chi-squared test was used to analyze the characteristics of the two groups according to the treatment response, and binary logistic regression analysis was performed to analyze the factors predicting a large SUVmax change. SPSS for Windows, version 24 (IBM Corp Armonk, NY, USA) was used for the statistical analyses. This study was approved by the Institutional Review Board (IRB) of Seoul St. Mary’s Hospital (IRB No. KC20RISI0365). The requirement for informed consent was waived by the IRB due to the retrospective nature of the study.

## Results

A total of 135 lesions were irradiated during the investigation period, and 27 sites in 12 patients who underwent PET-CT before and after treatment were included in the analysis. Patients with AML, acute lymphoblastic leukemia (ALL), and chronic myelocytic leukemia (CML) were included, of which eight received BMT before radiation therapy and four of them received TBI. Most of the patients had uncontrolled leukemia, and seven died during the follow-up period, of which five were recorded as related to infections. **Table 1** lists the characteristics of the 12 patients. In the 27 irradiated sites (**Table 2**), soft tissue was the most common (14 patients, 51.9%), followed by bone (11 patients, 40.7%), and organs (2 patients, 7.4%).

**Table 1:** Patient characteristics (N = 12).

Characteristic		N	%
Sex			
	Male	8	66.7
	Female	4	33.3
Age		Median 37.5 (14–52)	
	Adult	10	83.3
	Children	2	16.7
Disease			
	AML	5	41.7
	ALL	6	50.0
	CML	1	8.3
Previous BMT			
	No	4	33.3
	Yes	8	66.7
Previous TBI			
	No	4	33.3
	Yes	4	33.3
	Not-BMT	4	33.3
TBI dose			
	1200 cGy/6 fractions	1	8.3
	1320 cGy/8 fractions	3	25.0
Leukemia controlled			
	No	10	83.3
	Yes	2	16.7
Survival			
	Survival	5	41.7
	Death	7	58.3
Cause of death			
	Infection	5	41.7
	Disease progression	1	8.3
	Treatment-related complication	1	8.3

AML, acute myeloid leukemia; ALL, acute lymphoblastic leukemia; CML, chronic myelocytic leukemia; BMT, bone marrow transplantation; TBI, total body irradiation.

**Table 2:** Characteristics of the irradiated sites (n = 27).

Characteristic		N	%
Site			
	Soft tissue	14	51.9
	Bone	11	40.7
	Organ	2	7.4
Disease			
	ALL	12	44.4
	AML	13	48.1
	CML	2	7.4
RT volume		Median 43.06 (0.26–486.53)	
	< 40 cm^3^	13	48.1
	≥ 40 cm^3^	14	51.9
PreRT SUVmax		Median 7.47 (1.81–21.38)	
	<7.5	14	51.9
	≥7.5	13	48.1
RT technique			
	3D-CRT	9	33.3
	IMRT	18	66.7
RT fraction size			
	200 cGy/fraction	6	22.2
	250 cGy/fraction	7	25.9
	300 cGy/fraction	8	29.6
	500 cGy/fraction	6	22.2
BED10		Median 3125 (2400–5000)	
	< 3000 cGy_10_	12	44.4
	≥ 3000 cGy_10_	15	55.6

AML, acute myeloid leukemia; ALL, acute lymphoblastic leukemia; CML, chronic myelocytic leukemia; RT, radiotherapy; SUVmax, maximum standardized uptake value; 3D-CRT, 3-dimensional conformal radiotherapy; IMRT, intensity-modulated radiotherapy; BED10, biologically effective dose (alpha/beta ratio = 10).

Three out of 12 patients were first diagnosed with extramedullary chloroma, 8 patients developed myeloid sarcoma at their median relapses interval of 2.7 years after initial treatment, and 1 patient developed myeloid sarcoma as the disease progression during systemic treatment for median of 2.5 years. Twelve out of 27 irradiated sites were diagnosed by biopsy, and sites with difficult histological access were diagnosed by radiographic assumptions. All patients complained of symptoms due to the myeloid sarcoma lesions except for one site. Of the 27 treatment sites, 5 sites were treated with target therapy such as dasatinib, 3 sites were treated with reinduction treatment, and the other 19 sites were treated only with radiotherapy without concurrent therapy.

The SUVmax in PET-CT before radiotherapy was in the range of 1.81–21.38, with a median of 7.47, and the patients were classified into high and low SUVmax groups based on an SUVmax of 7.5. The radiation treatment dose was 200–500 cGy per fraction, and when converted to BED10, the median value was 3125 cGy_10_, ranging from 2400–5000 cGy_10_. The patients were classified into high and low irradiation groups based on a median BED10 of 3000.

**Fig 1:**
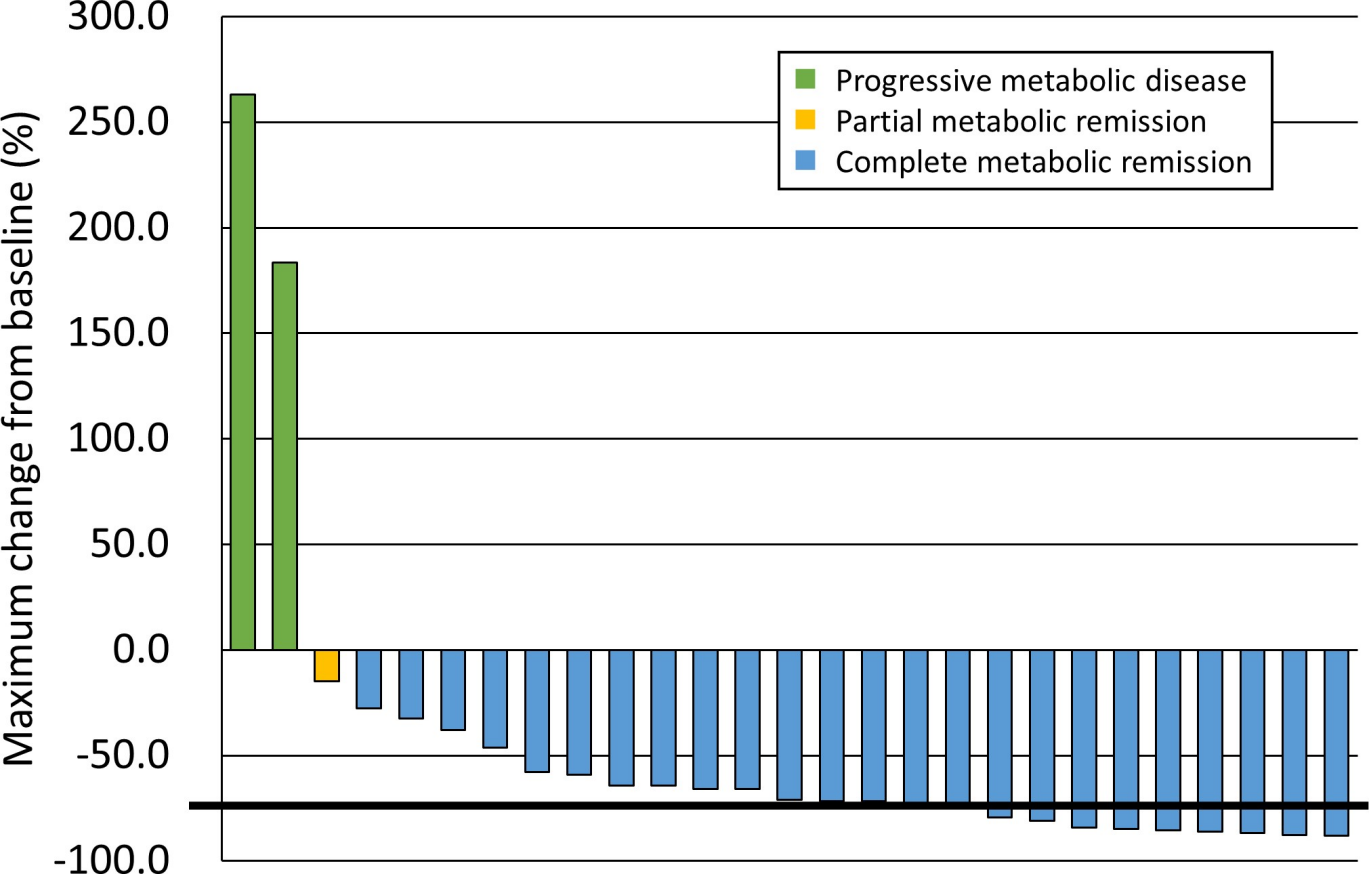
Waterfall plot of the maximum change in SUVmax. SUVmax, maximum standardized uptake value.

Evaluation of the response to PET-CT was performed at a median of 3.5 months (range, 1.9–7.3). The PET response after radiotherapy was classified as complete metabolic remission (CMR) at 24 sites (88.9%), partial metabolic remission (PMR) at one site (3.7%), and progressive metabolic disease (PMD) at two sites (7.4%). The change in SUVmax ranged from a decrease of 88.0% (-88.0%) to an increase of 263.0% (+263.0%), showing a median change of -70.9%. **Fig 1** shows a waterfall plot of the maximum change in the SUVmax. The two sites showing PMD were both arm muscles, and the pretreatment SUVmax values were 2.35 and 1.81, respectively (**Table 3**). In one ALL patient with extramedullary relapse, the follow-up SUVmax values of two irradiated lesions in PET-CT 6.4 months after completion of radiotherapy increased to 6.66 and 6.57, respectively. There was no PET response, but the patient’s symptoms improved. PET achieved CMR with additional chemotherapy, but the patient died from an infection related to chemotherapy.

**Table 3:** Characteristics of progressive metabolic lesions after radiotherapy.

Lesion number	1	2
RT site	Brachioradialis muscle	Flexor carpi ulnaris muscle
RT daily dose (cGy/fx)	200	200
RT fraction number	10	10
BED10 (cGy_10_)	2400	2400
RT technique	3D-CRT	3D-CRT
RT volume (cm^3^)	4.03	0.26
PTV margin (cm)	0.5	0.5
Pre-RT SUVmax	2.35	1.81
Post-RT SUVmax	6.66	6.57
Change of SUVmax (%)	+183.40	+262.98
Response EORTC	PMD	PMD
Symptom improvement	Yes	Yes
Salvage treatment	Further chemotherapy	Further chemotherapy
Survival	Death	Death

RT, radiotherapy; BED10, biologically effective dose (alpha/beta ratio = 10); 3D-CRT, three-dimensional conformal radiotherapy; PTV, planning target volume; SUVmax, maximum standardized uptake value; EORTC, The European Organization for Research and Treatment of Cancer; PMD, progressive metabolic disease.

Of the 26 sites that complained of symptoms during the follow-up period, symptoms did not improve in 7 sites, and symptoms improved in the other 19 sites. And there were recurrences in three sites, two sites with in-field recurrences and one site with marginal recurrence. The characteristics of the relapsed lesions are summarized in **Table 4**. The EORTC PET responses to radiotherapy were CMR in all three lesions and the decreases in the SUVmax after treatment ranged from 27.84–84.12. The relapse periods were 18.6, 8.7, and 6.7 months after the completion of radiotherapy. After recurrence, radiation therapy with a salvage aim was performed again, and the lesions were all controlled.

**Table 4:** Characteristics of recurrent lesions after radiotherapy.

Lesion number	8	15	21
Diagnosis	AML	ALL	ALL
Age at diagnosis	52	49	14
Sex	Female	Female	Male
Previous BMT history	Yes	Yes	No
RT site	Left lower extremity	Abdominal wall	Lumbar spine
RT daily dose (cGy/fx)	300	250	200
RT fraction number	10	10	10
BED10 (cGy_10_)	3900	3125	2400
RT technique	IMRT	IMRT	3D-CRT
RT volume (cm^3^)	17.39	123.32	3.77
PTV margin (cm)	0.5	1	-
Pre-RT SUVmax	5.46	14.74	6.06
Post-RT SUVmax	3.94	2.34	3.26
Change of SUVmax (%)	-27.84	-84.12	-46.20
Response EORTC	CMR	CMR	CMR
Relapse period (months)	18.6	8.7	6.7
Pattern of recurrence	In-field	Marginal	In-field
Systemic disease at recurrence	Uncontrolled	Uncontrolled	Controlled
Survival	Survival	Survival	Survival
Salvage treatment	re-RT	RT	re-RT

AML, acute myeloid leukemia; ALL, acute lymphocytic leukemia; RT, radiotherapy; BED10, biologically effective dose (alpha/beta ratio = 10); IMRT, intensity-modulated radiotherapy; 3D-CRT, three-dimensional conformal radiotherapy; PTV, planning target volume; SUVmax, maximum standardized uptake value; EORTC, The European Organization for Research and Treatment of Cancer; CMR, complete metabolic response; re-RT, re-irradiation.

The two lesions with disease progression and the three lesions with recurrence were defined as a treatment failure group, and the clinical factors were compared with the controlled groups and analyzed (**Table 5**). There was no significant difference in the radiation volume, site, concurrent therapy, and SUVmax before treatment, but the SUVmax after treatment showed a value of 2 or more in the treatment failure group, with a marginal statistical difference (*P* = 0.057). Although there was no significant difference between the radiation technique and the dose per fraction, the number of lesions irradiated with more than 3000 cGy_10_ based on BED10 was significantly greater in the treatment control group (*P* = 0.024). Characteristics of non-responding lesions including disease progression or relapse after radiotherapy were depicted in **S1 Table.**

**Table 5:** Comparison of clinical factors between treatment failure and controlled groups.

Characteristic		Treatment failure group	Treatment controlled group	*P*-value
		(n = 5)	(n = 22)	
Site				0.296
	Bone	1	10	
	Non-bone	4	12	
RT volume				0.114
	< 40 cm^3^	4	9	
	≥ 40 cm^3^	1	13	
PreRT SUVmax				0.163
	< 7.5	4	10	
	≥ 7.5	1	12	
PostRT SUVmax				0.057
	< 2	0	10	
	≥ 2	5	12	
RT technique				0.161
	3D-CRT	3	6	
	IMRT	2	16	
RT fraction size				0.185
	200–300 cGy/fraction	5	16	
	500 cGy/fraction	0	6	
BED10				0.024
	< 3000 cGy_10_	3	3	
	≥ 3000 cGy_10_	2	19	
Concurrent therapy				0.574
	Yes	2	6	
	No	3	16	

RT, radiotherapy; SUVmax, maximum standardized uptake value; 3D-CRT, three-dimensional conformal radiotherapy; IMRT, intensity-modulated radiotherapy; BED10, biologically effective dose (alpha/beta ratio = 10).

**Fig 2:**
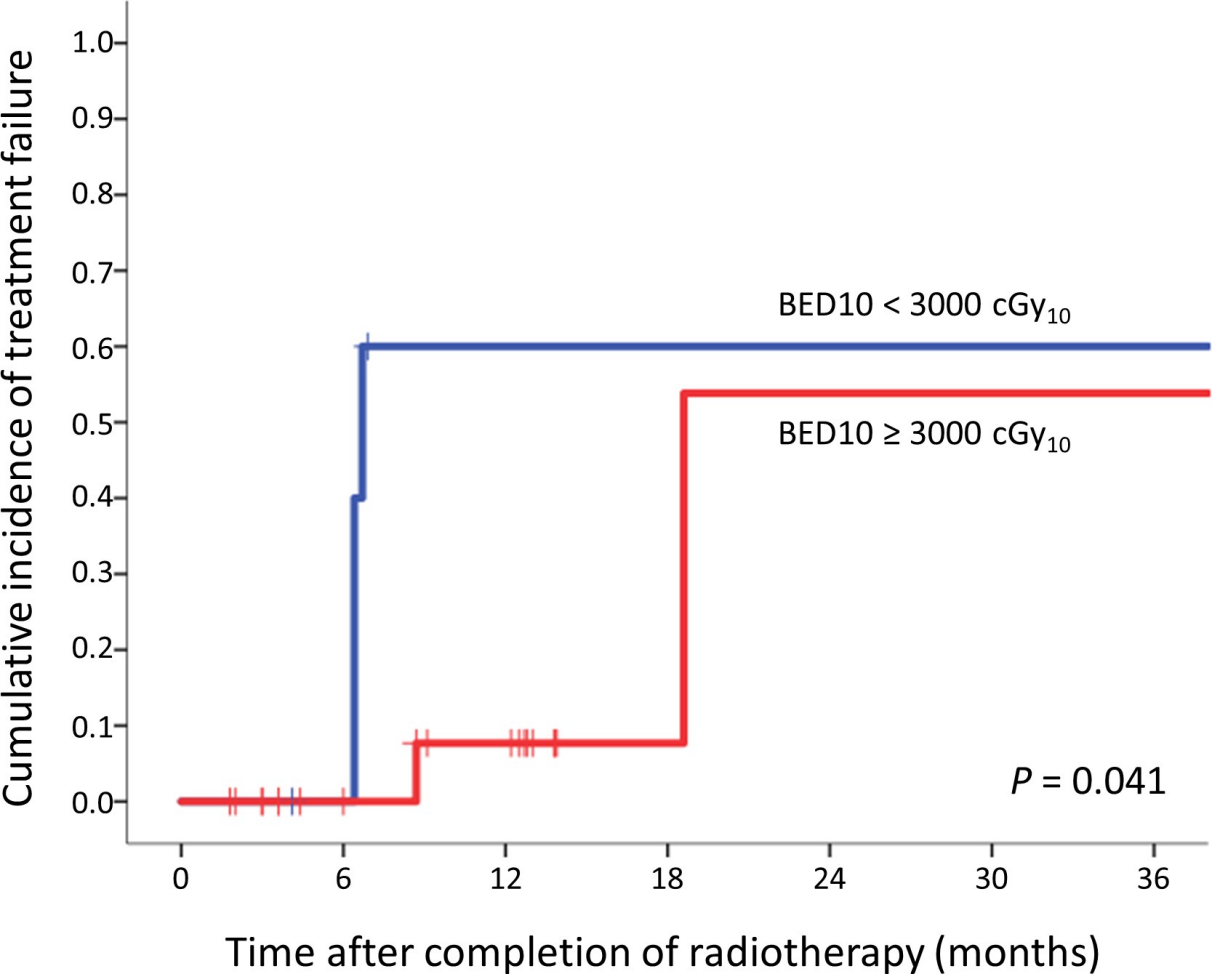
Kaplan-Meier curve for the cumulative incidence of treatment failure between radiotherapy dose ≥ 3000 cGy_10_ and < 3000 cGy_10_ groups.

The cumulative incidence of treatment failure was analyzed by using a log-rank test by dividing the group into groups with ≥ 3000 cGy_10_ and those with < 3000 cGy_10_. The Kaplan-Meier curve is shown in **Fig 2**, and there was a statistically significant difference between the two groups (*P* = 0.041). The 2-year treatment failure rates in two groups were 53.8% and 60.0%, respectively.

In this study, the median change in the SUVmax was -70%, and binary logistic regression analysis was performed to predict the changes in the SUVmax. In the univariate analysis, a high SUVmax of 7.5 or more before radiotherapy (*P* = 0.017) and further chemotherapy after radiotherapy (*P* = 0.018) were found to be significant factors. In the multivariate analysis, both of these factors were significant (further chemotherapy after radiotherapy, *P* = 0.026; pretreatment SUVmax, *P* = 0.027). Changes in the SUVmax decreased by more than 70% in cases when chemotherapy was not administered after radiotherapy and the SUVmax before treatment was higher than 7.5. The results are described in **Table 6**.

**Table 6:** Binary logistic regression analysis predicting reductions in SUVmax of ≥ 70% after radiotherapy.

Characteristic	Univariate	Multivariate analysis		
	*P*-value	Odds ratio	95% CI	*P*-value
RT site (Non-bone)	0.816			
RT volume (40 cm^3^)	0.335			
Further chemotherapy after RT (yes)	0.018	0.062	0.005–0.718	0.026
Pretreatment SUVmax (≥ 7.5)	0.017	13.862	1.350–142.341	0.027
RT dose (BED10) (≥ 3000 cGy_10_)	0.092			

CI, confidence interval; RT, radiotherapy; SUVmax, maximum standardized uptake value; BED10, biologically effective dose (alpha/beta ratio = 10).

## Discussion

Treatment for myeloid sarcoma-related leukemia is typically sensitive to chemotherapy and long-term remission has been reported when allogeneic hematopoietic stem cell transplantation was performed [10, 11]. Local treatment may be considered for organ invasion causing symptoms that may be life-threatening, such as to the spinal cord [5]. However, the risk of leukemia progression is high and the survival rate is not affected by local treatment alone [12]. Thus, radiation therapy is recommended for palliative purposes in combination with other treatments for symptomatic isolated lesions or patients who underwent previous transplantations [13].

Previous studies reported that the local recurrence of the treated lesion was low when radiotherapy was performed as a local treatment. Bakst et al. reported a 97% symptom relief effect for tumors irradiated with at least 20 Gy and showed a higher complete response rate compared to patients who did not receive radiation therapy [6]. Song et al. reported a symptomatic response of 85.7% when lesions were irradiated at 20 Gy in 10 fractions, and that small lesions less than 6 cm and soft tissue showed good complete remission rates [7]. In the present study, we evaluated the response of PET-CT according to EORTC PET criteria.

After radiotherapy, the SUVmax before and after treatment achieved CMR at 24 sites (88.9%) and PMR at one site (3.7%). In previous studies, PET-CT was reported to be useful as a diagnostic tool for extramedullary disease in patients with leukemia. In a recent prospective study, PET-CT had 77% sensitivity and 97% specificity for detecting extramedullary disease in AML patients [14]. In the present study, although the correlation between the SUVmax response and recurrence was difficult to analyze due to the lack of recurrence cases and the number of samples, we analyzed the factors affecting the recurrence and PMD showing treatment failure. In the results, treatment failures tended to be higher when the BED was low and the SUVmax increased after treatment. The results showed that the possibility of treatment control may be high when BED10 is irradiated with more than 3000 cGy_10_. Bakst et al. [6] proposed a schedule of 24 Gy in 12 fractions, which is BED10 2880 cGy_10_, suggesting that the higher dose in the current study could help control the PET response and recurrence.

**Fig 3:**
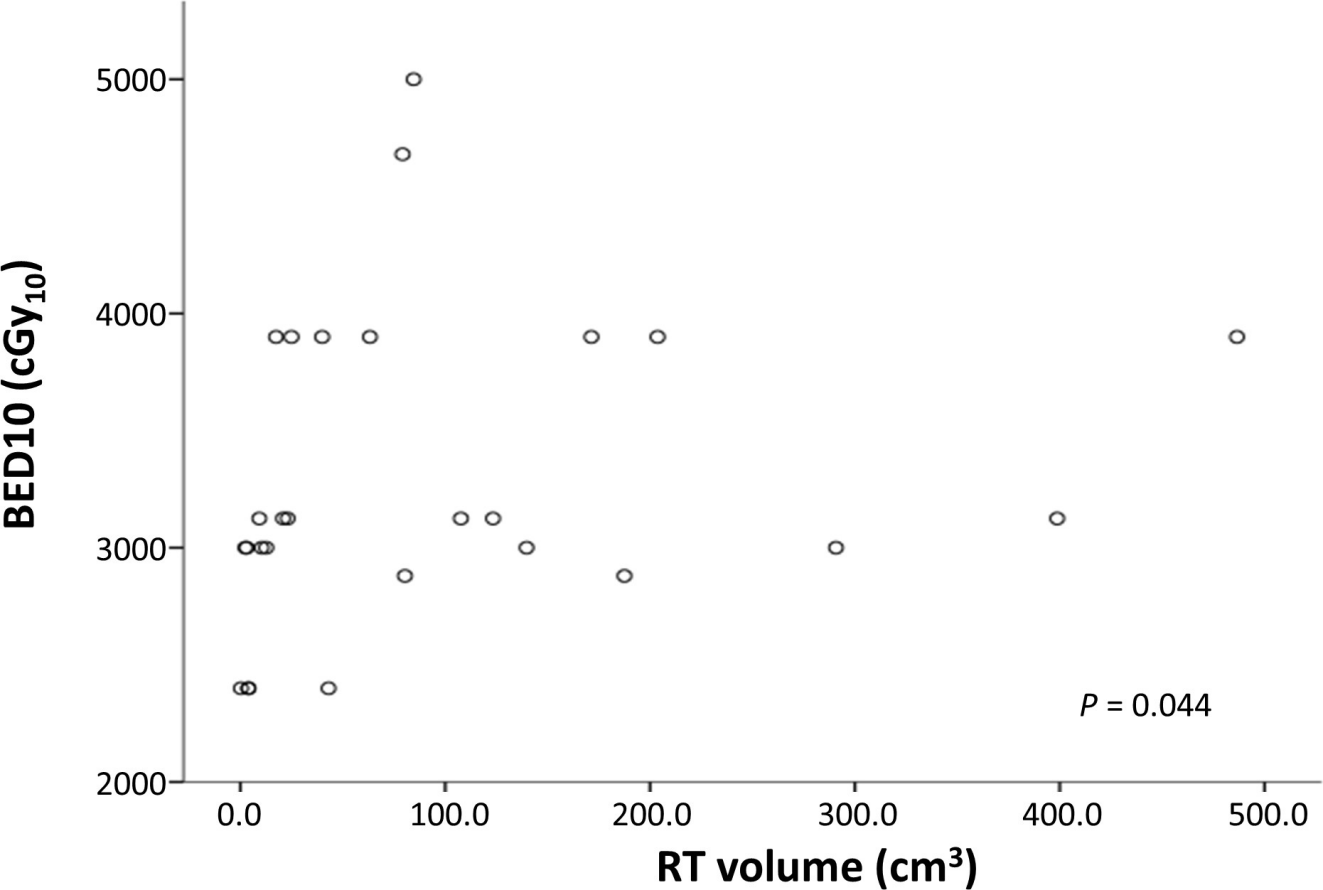
Scatter plot of the linear regression analysis of BED10 and irradiated volume. BED10, biologically effective dose with an alpha/beta ratio of 10.

Factors influencing the degree of reduction in the SUVmax were also analyzed in this study. A high SUVmax before treatment was identified as a factor influencing reductions in the SUVmax. This study differed from previous studies in that the degree of the decrease in the SUVmax after radiotherapy for myeloid sarcoma was analyzed. The degree of the decrease in the SUVmax was not related to the radiation treatment site, the irradiated volume, or the radiation dose. When reviewing the description of three recurrent sites, the radiation treatment site, volume, radiation dose, and SUVmax before treatment showed different characteristics (**Table 4**). Various disease entities (1 patient with AML and two patients with ALL) and 2 patients in non-adverse risk group showed that the radiotherapy response was not correlated with disease entity and adverse feature in leukemia. Further large-scale studies are needed to determine which clinical and radiologic factors are related to the recurrence or survival of myeloid sarcoma patients after radiotherapy. In addition, the radiation therapy doses were diversely distributed due to the limitations of the retrospective study. The BED10 showed a somewhat higher tendency as the radiation volume increased in the linear correlation analysis (*P* = 0.044), but a weak linear relationship (r = 0.391). The scatter plot (**Fig 3**) showed the heterogeneity of BED10, suggesting that a consensus of the schedule for the radiation treatment dose is necessary.

## Conclusion

In conclusion, radiotherapy for patients with myeloid sarcoma was a non-invasive local treatment modality, and 92.6% of patients achieved complete or partial metabolic responses after radiotherapy. The use of PET-CT before and after treatment is expected to be a useful tool for evaluating the response to treatment in addition to diagnostic purposes, and additional studies on PET-CT as an imaging test for treatment response and prognosis should be performed.

## Supporting information

S1 Table Characteristics of non-responding lesions after radiotherapy. (DOCX)
