# Supplementary material for: Analysis of PET parameters predicting response to radiotherapy for myeloid sarcoma
Source: PLoS One. 2021 Dec 20;16(12):e0261550. doi: 10.1371/journal.pone.0261550 (PMC8687562; doi:10.1371/journal.pone.0261550)
Supplement: S1 Table — (DOCX) [file pone.0261550.s001.docx]

| Lesion number | Diagnosis | Age at diagnosis | Sex | Cytogenetic characteristic | Adverse risk group | Timing of MS |
| --- | --- | --- | --- | --- | --- | --- |
| 1^,^ 2^*^ | ALL | 41 | Male | 47,XY,+2,-6,add(9)(p24),t(9;22)(q34;q11.2),add(10)(q24),+16 | Yes | At relapse |
| 8 | AML | 52 | Female | Normal | No | At relapse |
| 15 | ALL | 49 | Female | 45,XX,t(1;19;14;19)(q21;p13.1;q32;q13.1),-20 | Yes | At relapse |
| 21 | ALL | 14 | Male | 46,XY,t(1;10)(p34.3;q24.3) | No | At relapse |
| ^*^ Same patient  MS, myeloid sarcoma; ALL, acute lymphocytic leukemia; AML, acute myeloid leukemia. | | | | | | |

**S1 Table.** Characteristics of non-responding lesions after radiotherapy.
